# "Big" Food, Tobacco, and Alcohol: Reducing Industry Influence on Noncommunicable Disease Prevention Laws and Policies: Comment on "Addressing NCDs: Challenges From Industry Market Promotion and Interferences"

**DOI:** 10.15171/ijhpm.2019.30

**Published:** 2019-05-19

**Authors:** Belinda Reeve, Lawrence O. Gostin

**Affiliations:** ^1^ The University of Sydney Law School, Sydney, NSW, Australia.; ^2^ O’Neill Institute for National and Global Health Law, Georgetown University Law Center, Washington, DC, USA.

**Keywords:** Conflicts of Interest, Noncommunicable Disease, Law, Policy, Industry Influence

## Abstract

The food, tobacco and alcohol industries have penetrated markets in low- and middle-income countries (LMICs), with a significant impact on these countries’ burden of noncommunicable diseases (NCDs). Tangcharoensathien and colleagues describe the aggressive marketing of unhealthy food, alcohol and tobacco in LMICs, as well as key tactics used by these industries to resist laws and policies designed to reduce behavioural risk factors for NCDs. This commentary expands on the recommendations made by Tangcharoensathien and colleagues for preventing or managing conflicts of interest and reducing undue industry influence on NCD prevention policies and laws, focusing on the needs of LMICs. A growing body of research proposes ways to design voluntary industry initiatives to make them more effective, transparent and accountable, but governments should also consider whether collaboration with health-harming industries is ever appropriate. More fundamentally, mechanisms for identifying, managing and mitigating conflicts of interest and reducing industry influence must be woven into – and supported by – broader governance and regulatory structures at both national and international levels.

By 2040, low- and middle-income countries (LMICs) will experience a dramatic rise in disabilities, illness, and premature deaths from noncommunicable diseases (NCDs), such as cancer, diabetes, and cardiovascular or respiratory diseases.^1^ The epidemiological transition from communicable (and neonatal, maternal, and nutritional) diseases to NCDs in LMICs is driven by demographic trends, principally aging and economic development.^1^ Modifiable risk behaviours such as unhealthy diets, alcohol consumption, physical inactivity and tobacco use also fuel the rise of NCDs, driven in turn by hyper-urbanization, rapid economic growth, rising levels of disposable income, and other economic and socio-cultural changes associated with globalization.^1-3^ Beyond these drivers of NCDs lies the growing market penetration in LMICs by the food, tobacco and alcohol industries, accompanied by the aggressive marketing of these products – as described by Tangcharoensathien and colleagues.^2-4^

The growing burden of NCDs threatens not just individual and population-level health, but also national healthcare systems and social and economic development. This is recognized by the 2030 Sustainable Development Agenda: one of the targets for Sustainable Development Goal 3 (on ensuring health and promoting wellbeing) is reducing premature mortality from NCDs by one third by 2030.^5^ Innovative, cost-effective, and adequately funded healthcare systems are important to responding to the rise in NCDs in LMICs and for achieving the Sustainable Development Goals.^1^ Equally important are comprehensive national prevention strategies, including legal, regulatory, and fiscal capacities such as taxes on sugar-sweetened beverages (now introduced in over 40 countries worldwide),^6^ excise taxes to discourage alcohol purchasing and consumption, and mandatory warning labels on cigarette packets, alcoholic beverages, and unhealthy foods and drinks, with some countries currently exploring graphic warning labels for sugary beverages.^7-9^ Tangcharoensathien and colleagues aptly describe a major challenge faced by LMICs in robustly regulating multinational corporations, namely industry’s undue influence on law and policy-making.^4^ The authors in this commentary discuss strategies for ameliorating industry interference and conflicts of interest in the design and implementation of NCD prevention measures.

## Industry Influence on NCD Law and Policy-Making

The large, multinational companies that dominate the processed food, alcohol, and tobacco industries are described as “vectors” of the NCD epidemic.^2^ Their most obvious role as disease vectors lies in the manufacture, sale and marketing of products that are harmful to health. Beyond this, the alcohol, tobacco and food industries exert a powerful influence on laws and policies that address the behavioural risk factors for NCDs.

Tangcharoensathien and colleagues describe key tactics used by “Big” Food, Tobacco, and Alcohol to weaken or forestall NCD prevention measures, including litigating against strong public health interventions, using front groups to counter public health recommendations, funding and influencing research favourable to industry interests, and gaining preferential access to policy-making processes by building relationships with health leaders and legislators.^4^ Industry uses a range of other tools to influence policies harmful to its economic interests, including pre-emption of state, provincial, or local laws to prevent NCDs; adopting self-regulation or voluntary measures to stave off stronger statutory regulation^9,10^; and shaping public dialogue on NCD causation, promoting discourses of individual choice, personal responsibility, and the “Nanny State,” while resisting science-based interventions that facilitate shared responsibility and government action on NCDs.^11^ In addition, industry actors tend to set their own targets or metrics for what they are willing to achieve in NCD prevention, rather than adopting more stringent best-practice targets recommended by national governments or the World Health Organization (WHO). As the authors note, all three industries use a “playbook” of similar tactics to avert effective public health interventions,^12^ with a growing body of evidence demonstrating their use in emerging as well as developed countries.^2,13,14^

## Should Policy Actors Engage With Health Harming Industries?

The negative influence of health-harming industries on NCD prevention raises questions about whether public officials should ever engage with industry and, if they do, how to manage conflicts of interest and undue industry influence. The WHO *Framework Convention on Tobacco Control* strictly forbids engagement with the tobacco industry.^15^ States parties must take steps to protect tobacco policy-making from industry interference (FCTC, article 5.3). Should the same strict standards apply to Big Food and Alcohol?

Governments often explicitly or tacitly encourage self-regulation by the food and alcohol industries (including by doing nothing or remaining complicit), or join with industry actors in collaborative initiatives and public-private partnerships that have NCD prevention objectives. Yet a significant body of evidence shows that self-regulatory or collaborative initiatives have been largely ineffective in achieving public health objectives,^16^ and some researchers question whether credible and effective engagement with the food and alcohol industries is ever possible. These authors point to an inherent conflict of interest between NCD prevention and the profit-making imperative of businesses selling unhealthy products, as well as the often-significant power imbalance between large, multinational companies and national governments.^11,17^

In relation to the food industry, a more nuanced position may be to identify and assess the variety of actors and modes of engagement that are possible, rather than to propose a blanket ban on all forms of engagement. WHO’s guidance document on preventing and managing conflicts of interest in nutrition policy-making and implementation lists a six-step process of risk assessment, balancing, and mitigation.^18^ The six steps include: clearly identifying the public health nutrition goal to be achieved through engagement with non-state actors; profiling, due diligence and risk assessment (including assessing non-state actors against exclusionary criteria); balancing the risks and benefits of engagement; risk management (including identifying the risk profile of external actors and the form that engagement would take, and introducing mitigation measures where engagement is considered appropriate); monitoring, evaluation and accountability; and transparency and communication. While this document provides a detailed pathway for risk management and mitigation, for many in civil society, any engagement with the food and alcohol industries is fraught with conflicts that cannot be managed.

In relation to the alcohol industry, a stronger case can be made that engagement is never appropriate, given the status of alcohol as “no ordinary commodity,” that alcohol cannot be said to be crucial to human health and existence in the same way as (some) foods, and the lack of any evidence that collaboration with the alcohol industry leads to public health benefits.^19,20^

## Managing Conflicts of Interest and Holding Industry Accountable

A growing body of research documents ways to make engagement with industry more effective, accountable and transparent.^10,21,22^ If governments plan to work with industry on self-regulatory or voluntary initiatives, they must take firm measures to avoid harmful or unjust results. Table describes specific recommendations for improving the effectiveness and accountability of such initiatives (adapted from Reeve^23^ and WHO^18^). In addition, strong government leadership is essential: governments should lead processes of policy development and create a policy or legislative framework that sets targets for action against which progress can be measured and policy coherence maintained.^24^ Such legislation could also include principles to govern the implementation of public-private partnerships, including the clear identification and mitigation of any conflicts of interest.^24^ Further, governments should (credibly) threaten legislation if voluntary or collaborative initiatives fail to meet public health objectives.^22^

**Table:** Table. Recommendations for Creating Effective, Transparent and Accountable Forms of Voluntary or Collaborative Initiatives With Industry

**Component**	**Recommendation**
Developing objectives/policies	Consultation with a broad range of interests.
Objectives	Clear, measurable objectives set by government, against which the scheme’s success or failure can be assessed.
Terms and conditions	Key definitions are clear, conditions and roles are set by government, and are expansive or demanding enough to meet set objectives.
Administration	Fair and transparent administration by an accountable, independent body, with the roles and responsibilities of each member clearly described.
Monitoring	A comprehensive, transparent, and independent monitoring system that can be used to evaluate the initiative and which includes baseline data, as well as a set of measurable, time-bound process and outcome indicators. The results of monitoring and evaluation exercises are made publicly available, to enhance transparency and accountability to external stakeholders. Public reporting should include information on the outcomes of the initiative.
Enforcement	A wide range of enforcement options are available, including both incentives and deterrents, as well as an effective, accessible complaints-handling mechanism where appropriate.
Review	Regular, independent reviews of the scheme’s operation, using baseline data and performance indicators, with reports from reviews made publicly available.

Civil society organizations and non-government watchdog groups play a critical role in advocating for strong NCD prevention measures and in monitoring both industry and government efforts, thereby holding both sectors accountable for commitments and responsibilities. Voluntary or collaborative initiatives could potentially be strengthened by enabling non-governmental organisation participation on an equal footing with government and industry, and by granting civil society actors powers to monitor and enforce such initiatives.^25^

Tangcharoensathien and colleagues argue that a comprehensive strategy for managing conflicts of interest requires more than just the careful design of initiatives. Addressing industry influence and ensuring the effectiveness of voluntary or collaborative measures requires strengthening governance institutions more broadly and ensuring that political and regulatory processes are democratic and free from corruption or capture.^4^ At a national level this could include legislation requiring the disclosure of information by lobbyists in the form of a register, recusal of public officials from functions where they have a conflict of interest,^18^ and ensuring adequate funding for government institutions and regulatory agencies.

Emerging countries in particular may require technical and financial support from national and international actors to assist them in managing industry influence or interference with NCD prevention measures. For example, the beverage industry has invested substantial funds in defeating sugar-sweetened beverage taxes through litigation, which may have a “regulatory chill” effect, particularly in LMICs without the legal or financial resources to defend such challenges.^26^ Understanding how to defend these cases and having the financial capacity to do so can help ward off industry influence.^26^ This is illustrated by Bloomberg Philanthropies’ (and partners’) support for the Uruguayan government in successfully defending its tobacco control laws against a legal challenge by Philip Morris International under international trade law.^27^ Support for LMICs could be enabled by the sharing of best practice between countries on defending litigation and on managing conflicts of interest.^26^ Litigation and other complaints-handling mechanisms can also be used by countries themselves to hold industry accountable for the harms caused by their products and to vindicate health-related rights, as with tobacco litigation in the United States.^9^

## The Importance of International Governance Frameworks

International governance can help or hinder governments in managing conflicts of interest and eliminating inappropriate industry influence on policy-making. To date, the WHO has taken a vague and inconsistent stance on managing conflicts of interest and engaging with industry.^28^ A strong stance is evident in the WHO’s *Framework Convention on Tobacco Control*; in comparison, the *Strategy on Diet, Physical Activity and Health* envisages industry participation in activities related to diet, physical activity and health (“thus formalising institutional conflicts of interest”),^28,29^ while the *Global Strategy to Reduce Harmful Use of Alcohol* says little on conflicts of interest and encourages “economic operators” to consider voluntary or self-regulatory action.^30^

Recent documents evidence a more cautious stance, as with the WHO’s guidance document on managing conflicts of interest in nutrition policy (discussed above),^18^ and its *Framework on Engaging with Nonstate Actors*.^31^ The WHO *Global Action Plan for the Prevention and Control of NCDs 2013-2020* also requires Member States to protect NCD prevention policies from undue influence by vested interests and to acknowledge and manage real, perceived or potential conflicts of interest.^32^ However, there is scope to further clarify and operationalise the WHO’s stance on engaging with health-harming industries,^28^ particularly in relation to the alcohol industry.

Human rights instruments may support the efforts of national governments to reduce industry influence on law- and policy-making. Most countries have ratified at least one international agreement that recognizes the right to health and/or other health-related rights (eg, the right to food), such as the International Covenant on Economic, Social and Cultural Rights.^9^ Under this international treaty, States Parties have an obligation to respect, protect and fulfil the right to health, which includes preventing corporate human rights violations.^9^ Treaty monitoring bodies have recommended that states take legal and policy measures to address NCDs in reports issued as part of country periodic review processes; such recommendations could strengthen the position of countries counteracting litigation from food, tobacco or alcohol companies.^9^

In contrast to human rights legislation, international trade and investment agreements potentially restrict the ability of states to protect NCD laws and policies from industry influence. They also provide industry actors with an avenue to provide input into public health policy-making and to challenge legislation via national and international litigation.^9,33^ For example, Philip Morris unsuccessfully challenged Australia’s tobacco plain packaging laws under an international investment agreement between Australia and Hong Kong (and also challenged the constitutionality of these laws in Australia’s domestic court system). Such cases illustrate that international investment agreements may expose countries to legal liability when introducing new laws on NCD prevention, risking “regulatory chill.”^34^ Trade liberalization also fosters foreign direct investment by companies such as Coca-Cola in emerging markets in LMICs, which is anticipated to increase consumption of unhealthy food and beverages, and to create tensions between government measures to encourage such investment and NCD prevention policies.^34^

It is crucial for the public health community to be active in trade and investment policy processes, and for national governments to avoid agreements that overly constrain their ability to introduce public health laws and policies.^34^ Other helpful measures include strengthening the global governance of NCDs, eg, by the WHO creating new, legally binding standards on nutrition and reducing harmful consumption of alcohol (which could be used as a reference in trade dispute arbitration), as well as specific language in new trade and investment agreements requiring consideration of WHO-approved action plans or recommendations in any dispute with NCD prevention implications.^33^

## Countering Industry’s Pervasive Influence on NCD Policies

A large body of research documents the ways in which the food, tobacco, and alcohol industries attempt to influence law and policy-making to prevent NCDs, including in LMICs – a rapidly growing market for health-harming products. Researchers and international organisations such as the WHO are paying increasing attention to ways in which conflicts of interest can be avoided or managed, as well as reducing industry influence on policy-making. Active consideration should be given to whether engagement with food and alcohol industry actors should be excluded altogether. Where governments do engage with industry, self-regulatory or collaborative initiatives can be designed to improve their transparency, accountability, and effectiveness. However, a comprehensive approach to reducing inappropriate industry influence requires that rigorous conflicts of interest rules must be woven into – and supported by – broader governance structures at national and international levels.

The undue influence of “Big” Food, Alcohol and Tobacco is far from a theoretical matter. These industries are responsible for millions of preventable deaths and countless suffering. If multinational corporations fail to exercise social responsibility, it is incumbent on governments – and global health bodies – to compel them to do so.

## Ethical issues

Not applicable.

## Competing interests

Authors declare that they have no competing interests.

## Authors’ contributions

BR led the initial drafting of the article and both BR and LG edited the article, provided written contributions, and reviewed the final version.

## Authors’ affiliations

^1^The University of Sydney Law School, Sydney, NSW, Australia. ^2^O’Neill Institute for National and Global Health Law, Georgetown University Law Center, Washington, DC, USA.
